# Equivalent Neural Network Optimal Coefficients Using Forgetting Factor with Sliding Modes

**DOI:** 10.1155/2016/4642052

**Published:** 2016-12-13

**Authors:** Karen Alicia Aguilar Cruz, José de Jesús Medel Juárez, Romeo Urbieta Parrazales

**Affiliations:** Centro de Investigación en Computación, Instituto Politécnico Nacional (CIC-IPN), Avenida Juan de Dios Bátiz, Esq. Miguel Othón de Mendizábal, Col. Nueva Industrial Vallejo, Delegación Gustavo A. Madero, 07738 Ciudad de México, Mexico

## Abstract

The Artificial Neural Network (ANN) concept is familiar in methods whose task is, for example, the identification or approximation of the outputs of complex systems difficult to model. In general, the objective is to determine online the adequate parameters to reach a better point-to-point convergence rate, so that this paper presents the parameter estimation for an equivalent ANN (EANN), obtaining a recursive identification for a stochastic system, firstly, with constant parameters and, secondly, with nonstationary output system conditions. Therefore, in the last estimation, the parameters also have stochastic properties, making the traditional approximation methods not adequate due to their losing of convergence rate. In order to give a solution to this problematic, we propose a nonconstant exponential forgetting factor (NCEFF) with sliding modes, obtaining in almost all points an exponential convergence rate decreasing. Theoretical results of both identification stages are performed using MATLAB® and compared, observing improvement when the new proposal for nonstationary output conditions is applied.

## 1. Introduction

Artificial Neural Networks (ANNs) are computational models based on Biological Neural Networks (BNN) synapses description. Biological neurons include a fire function after concatenating the neuron inputs with innovative conditions, given by external stimuli and excitation signals. The answer of this function is transmitted and manipulated, obtaining interconnections with other neurons and integrating a complete network. On the other hand, ANN models are famous because of having the ability to learn and adjust their parameters dynamically, adding factors compound by corrections, or different combining techniques including expert systems [1].

Instead of investing in high computational resources to represent ANN by adding factors to adjust its hidden gains, in [2] are proposed three model approximations allowing the identification selecting, in some sense the gains that the neural net requires, and showing three different representations of ANNs, considering characteristics that make them ideal for modelling and identification and also indicating that nonlinear models could be interpreted as an ANN with specific properties. Unfortunately, the ANNs by themselves have poor performance in identification and estimation tasks when considering nonlinear systems and are not adequate to accomplish online requirements due to their complex algorithms, which are usually based on stable and invariant conditions [3].

A solution for these restrictive conditions is the combination of ANNs with traditional methods and others, such as sliding modes (SM), probability, or fuzzy logic (FL), improving the ANNs performance in convergence sense [4–6]. Nevertheless, the combination of several algorithms also increases its complexity, giving place to a higher computational cost.

The Equivalent Artificial Neural Network (EANN), developed in [7], is a representation that considers the linearization of a multiple input-single output (MISO) system and is useful for cases where time is an important factor, applying different techniques to adjust their gains according to a reference, even with difficulty in modelling external perturbations. In general, any ANN could be reduced to a simpler equivalent model (EANN) integrated by multiple inputs that interact with a set of weights combined in some sense giving a final output [7, 8].

**Figure 1:**
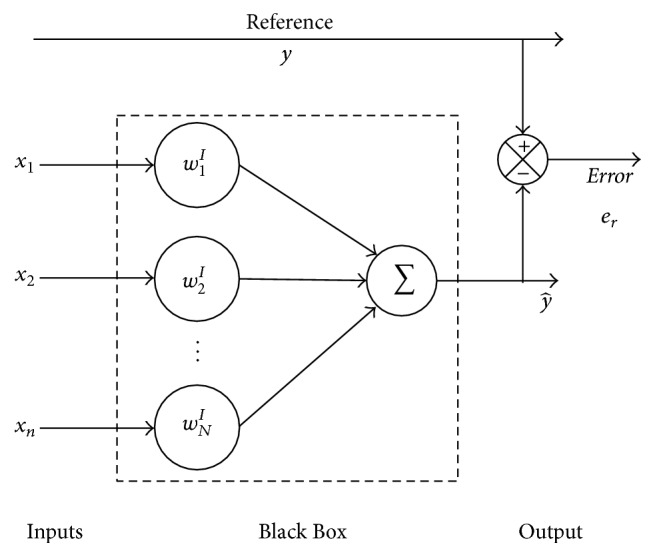
Equivalent Artificial Neural Network viewed as a Black Box (BB).

In [9], different adaptive algorithms are presented, where the desired signal is compared to an output having a correction error. The diagram representations are accomplished with the EANN description. Therefore, it is possible to consider the EANN through the Black Box (BB) concept as a MISO system by having the ANN inputs as the BB input vector and the output signal as the one to be compared to the nonstationary reference [1, 8, 10]. Then, the dynamic weights or parameters are calculated, using them instead of those that are random traditionally assigned, whose unique restriction is a preestablished range. These considerations generate a fewer calculation cycles obtaining the desired nonstationary output and reducing the computational cost.  Figure 1 shows the analogy between the EANN and the BB and compares both answer signals through the error.

Recursive and traditional identification, for example, through the Least Square Method (LSM) or Kalman Filter (KF), has good results on average as long as the assigning of the initial conditions is adequate. Nevertheless, their performance is reduced when a point-to-point approximation is developed. As the initial weights assignation is not enough, including a forgetting factor (FF) in the estimation process enables the minimization of the convergence error in almost all points [11].

FFs have been used as constants or linear functions, improving the parameter estimation; now the question is what would happen if the system conditions vary significantly from the point where a constant or linear FF was designed? Does it stop being useful? In this sense, different approaches use individual coefficients for particular system evolution times, changing along intervals. Others apply the exponential function to the evolution or sampling time as the argument, presenting a faster exponential convergence to the reference, but only good for smooth changes [12].

The equivalent EAAN model requires the weights estimation with time-varying conditions and considering smooth movements, as a first application. Nevertheless, in many cases, the BBs have nonstationary conditions with ranges that exceed the smooth ideal conditions; that is, the first two probability moments are bounded by distribution functions, respectively, without solving movement tracking tasks, so that there are researchers continuing proposing different adaptive techniques.

In spite of all combinations developed, fortunately in this paper we propose a novel estimation technique combining three traditional tools: (a) the estimation using LSM with instrumental variable, applying the reference signal and the convergence error (built by the difference between the reference and the EAAN output answer) and its sign, (b) the sliding surface based on error properties that allow developing a new evaluation strategy, minimizing the convergence error in less time than the traditional LSM [13, 14], and (c) an innovative exponential FF (EFF) applying traditional SM. The strategy considered is over the traditional estimation because both the calculation of EFF and the construction of the new estimation are made by using SM, a combination that allows for tracking nonstationary weights or parameters.

## 2. Optimum Weight Values

For making an analytical analysis of the EANN, its elements can be considered as vectors, so that the number of variables is conserved and applicable for any proposition. From Figure 1, the input signals *u*_*k*_ will be represented by the vector *x*_[*N* × 1]_, the weights are calculated as the vector *A*_[1 × *N*]_, and the output signal will be seen as *y*_[1 × 1]_ [15]. These considerations agree with a first-order MISO system defined as

(1) $$\begin{array}{c} y_{k}=\left[\;A\;\right]\left[\;x_{k}\;\right], \end{array}$$

which could be solved by using the expected value in a probabilistic sense. From (1), where the input vector and the desired signal are valid for a specific instant of time *k*, we clear *A*. Thus, we obtain ${\hat{A}}_{k\left[1\times N\right]}=E\left\{y_{k}{\left[x_{k}\right]}^{T}\right\}{\left[E\left\{\left[x_{k}\right]{\left[x_{k}\right]}^{T}\right\}\right]}^{+}$ and its discrete form:

(2) $$\begin{array}{c} {\hat{A}}_{k\left[1\times N\right]}=\left(\;k^{-1}\sum y_{k}x_{k}^{T}\;\right){\left(\;k^{-1}\sum x_{k}x_{k}^{T}\;\right)}^{+}. \end{array}$$

The results of ${\hat{A}}_{k}$ are applied into the output identification ${\hat{y}}_{k}$ and compared to the reference signal *y*_*k*_, obtaining the error $e_{rk}≔y_{k}-{\hat{y}}_{k}$, whose functional *J*_*n*_≔*E*{*e*_*rk*_} would tend to zero when ${\hat{A}}_{k}$ is optimum.

The description is for the optimum vector coefficients on average so any correction through feedback is not necessary; nevertheless, there is a need to express the solution recursively and to apply it to the equivalent model system, observing the answer evolution through time.

First, we define *P*_*k*[1 × *N*]_ = *k* ^−1^∑*y*_*i*_ *x*_*i*_^*T*^ and *Q*_*k*[*N* × *N*]_ = *k* ^−1^∑*x*_*i*_ *x*_*i*_^*T*^ with their recursive forms represented by the following, respectively:

(3) Pk1×N=k−1ykxnT+k−1Pn−1,

(4) QkN×N=k−1xkxkT+k−1Qk−1.

**Figure 2:**
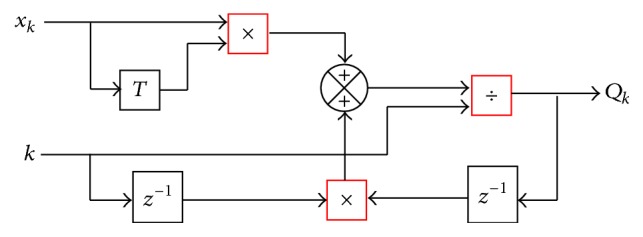
Block diagram for obtaining *Q*_*k*_, recursively.

**Figure 3:**
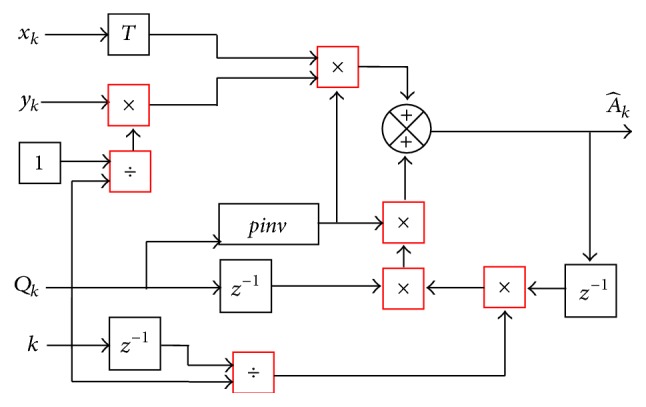
Block diagram for obtaining ${\hat{A}}_{k}$, the weight vector, recursively.

Substituting (3) and (4) into (2) and then expressing it in terms of *Q*_*k*_, whose block diagram is shown in Figure 2, we determine the ${\hat{A}}_{k}$ vector, the term whose description is in the following and Figure 3:

(5) $$\begin{array}{c} {\hat{A}}_{k\left[1\times N\right]}=\left(\;k-1\;\right)k^{-1}{\hat{A}}_{k-1}Q_{k-1}Q_{k}^{+}+k^{-1}y_{k}x_{k}^{T}Q_{k}^{+}. \end{array}$$

## 3. Exponential Forgetting Factor (EFF)

The system response when using (5) is adequate for cases that need an average approximation, meaning the system has constant parameters. Nevertheless, as we do not know what the weights are or if they suffer any change through the system evolution, it is necessary to improve the estimation technique by creating a more robust procedure.

In [16], we proposed to use an FF for nonnormalized Least Mean Square Algorithm (NLMSA) to improve it. Meanwhile, in [17] is used a deterministic FF to achieve an Optimized Convergence Rate (OCR).

The FF is used to reduce the influence of past information for the calculation of new parameters, obtaining a renewed approximation and braking with the convergence to the media value of the complete process. In [18] it was suggested to use an equivalent ANN model and the sliding modes (SM) in combination with an FF, which gradually reduces the influence of past data. On the other hand, in [19] it is indicated that the FF value should be between zero and one, so that when it is closer to zero, it discards the old data faster, making the response more sensitive to the new data; the opposite happens when the FF is closer to one.

To obtain an optimum coefficient which allows a better response, where the output follows the variations generated in the reference, we propose a nonconstant exponential FF (NCEFF), as the following indicates:

(6) $$\begin{array}{c} {eff}_{k}^{e_{rk}}=\mathrm{sign}\left(\;{\hat{A}}_{k}\;\right)e^{\left(\mathrm{sign}\left({\hat{A}}_{k}\right)e_{rk}\right)}, \end{array}$$

whose properties depend on the error *e*_*rk*_, considered as an innovation process, and are based on the calculation of ${\hat{A}}_{fk}$ and its sign [13, 14]. The sign function gives the SM sense and allows for converging not only to positive parameters, so that it is necessary to use it as an additional tool.

Applying (6) in combination with (5), and its sign function, we obtain (7), which is an innovative way to calculate a new weight parameter ${\hat{A}}_{fk}$, through an improved technique.

(7) $$\begin{array}{c} {\hat{A}}_{fk}=\left(\;{\hat{A}}_{k}+{eff}_{k}^{e_{rk}}\;\right)-\mathrm{sign}\left(\;{\hat{A}}_{k}\;\right). \end{array}$$

## 4. Simulation and Results

We developed the simulations in MATLAB integrating both analysing its performances with respect to a reference signal and substituting (5) and (7) into (1). The comparisons give us the idea of how the new estimation technique improves the original estimation presented in [7] for the EANN. In graphics, the estimation named as “optimum” is made using (5) while the “EFF” estimation includes the implementation of (7).

**Figure 4 fig4:**
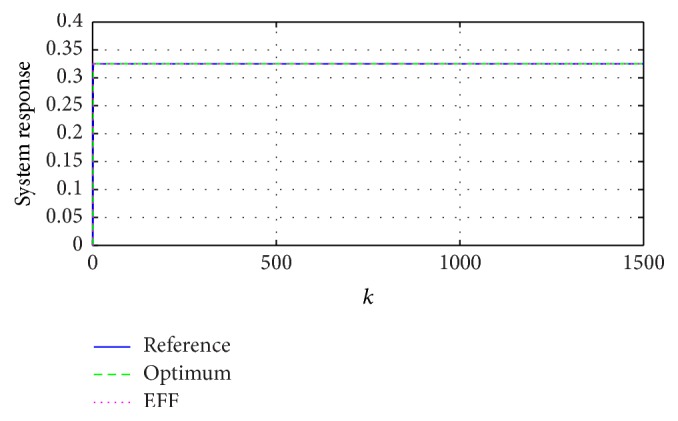
System response: reference with invariant parameters, estimations through the optimum coefficient (optimum), and the exponential forgetting factor (EFF).

Beginning with the simplest reference, a signal with invariant parameters and no external noise is possibly seen as a constant function *y*_*k*_ = [*A*], where {*A*} ∈ *R*_[−1,1]_ is constant.  Figure 4 compares the reference with the responses that use the estimated parameters through (5) and (7).

**Figure 5 fig5:**
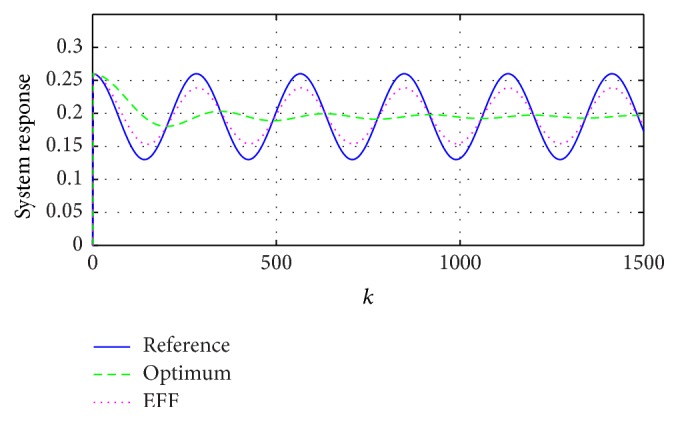
System response: reference with variable parameters, estimations through the optimum coefficient (optimum), and the exponential forgetting factor (EFF).

As a second test, Figure 5 presents the response when the system has variable parameters without external noise, describing how both approaches behave and their scopes; in this case, the reference comes from *y*_*k*_ = [*A* + *w*_*k*_][*x*_*k*_], where *w*_*k*_{*w*_*k*_}⊆*N*(*μ*_*w*_*k*__, *σ*_*w*_*k*__^2^ < *∞*) modifies the vector parameters.

The stationary system response gives an idea of how the estimation could be made and is included in the proposed model. Comparing both, the difference generated affects the evolution model in some sense because the parameters are function of the convergence error.

The main objective of the present paper is to build a special stochastic estimation, which affects the output model system, seeking a better convergence rate, where *y*_*k*_ = [*A* + *w*_*k*_][*x*_*k*_] + [*v*_*k*_], where *v*_*k*_ is a random variable {*v*_*k*_}⊆*N*(*μ*_*v*_*k*__, *σ*_*v*_*k*__^2^ < *∞*).

**Figure 6:**
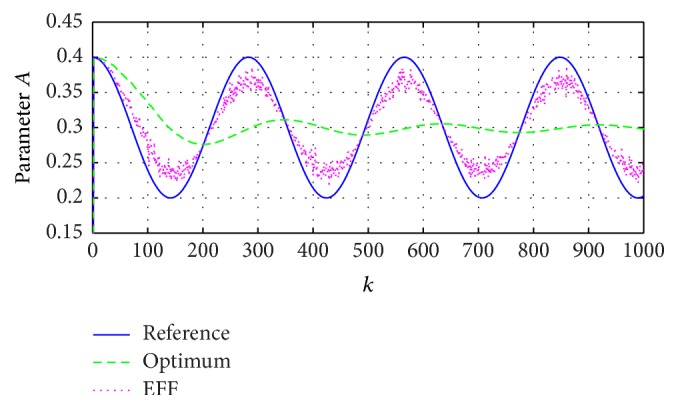
Comparison of the ${\hat{A}}_{k}$ parameter values: reference signal parameters, estimated through optimum coefficient ${\hat{A}}_{k}$(optimum), and the exponential forgetting factor ${\hat{A}}_{fk}$ (EFF).

**Figure 7:**
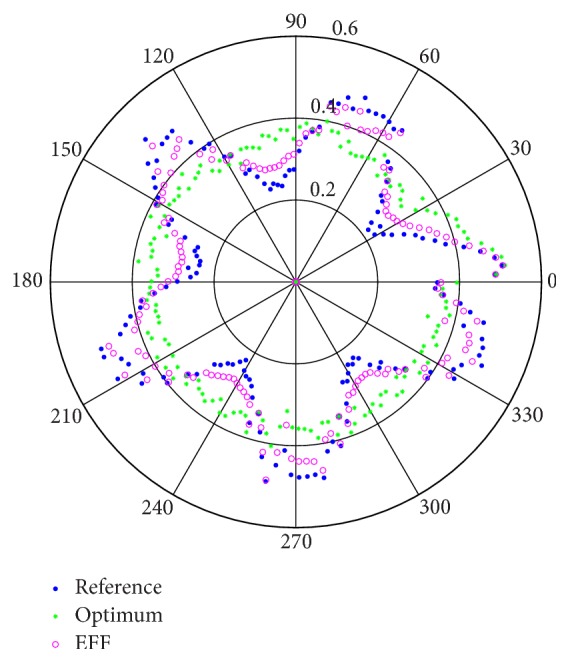
Polar graphic with a trajectory comparison of the approaches of the estimation methods: optimum coefficient (optimum) and optimum coefficient with forgetting factor (EFF) to a stochastic reference signal.

Now, for the third simulation, we will suppose being able to measure the internal parameters; nevertheless, they are only as an illustrative consideration. Then, Figure 6 compares the unknown system parameters to the estimated ones, and Figure 7 represents the approaches to reach a reference signal, viewed as a polar trajectory.

## 5. Application Example

**Figure 8:**
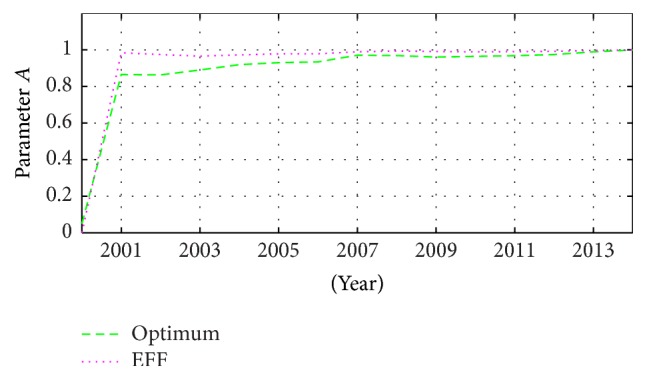
Estimation of the parameters that define the Passenger Traffic, within a period, in Mazatlán, México.

The previous considerations could be applied, improving, for example, the resource distribution of a touristic place according to the season current situation, calculating the incomes, the tourism charge, and economic events [20, 21] oscillating in a period of time > 10 years as shown in Figure 8.

**Figure 9:**
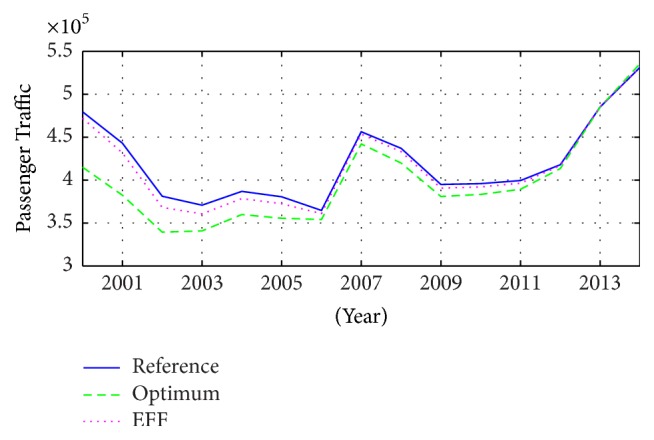
National Passenger Traffic in Mazatlán, México, and its estimations through the optimum coefficient (optimum) and the exponential forgetting factor (EFF).

These parameters help to determine the passenger numbers going to a touristic place, according to the information from previous years [22].  Figure 9 shows the behaviour of this phenomenon and its identification, describing the nonstationary tendency through a stochastic estimation with sliding modes, describing a nondeterministic situation, point-to-point.

**Figure 10:**
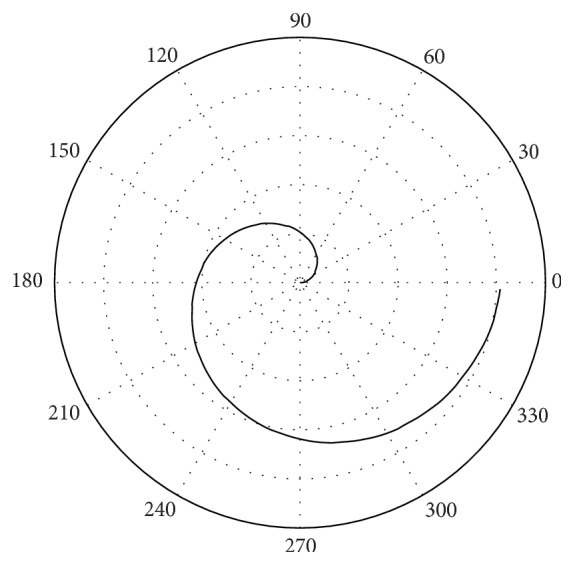
Entropy representation of a stochastic process.

Then, Figure 10 presents the entropy rises of incoming passengers process viewed in polar form, in the same period of years.

## 6. Conclusions

The present paper demonstrates the advantages and limits of a classic approximation through stochastic system when using the expected value, which, as shown in Figures 3 [Fig fig4] [Fig fig5]–6, has a good performance in a distribution sense.

Nevertheless, when more precise results are needed, it is necessary to add a correction factor in order to track the parameters that are far from the average, to give a better output response for systems with stochastic characteristics (Figures 7 and 9), such as the proposed dynamic exponential forgetting factor (EFF).

The use the Euler number and the sign function (SF) improved the complex trajectory, as seen in Figure 7, because the implementation of error properties affects the exponential forgetting factor (NCEFF) and sliding modes.

The SF allows the convergence towards the parameters that are not only above the reference but also below this, while the absolute function allows the conversion to only one side of them. In addition, the error used determines the EFF actualized point-to-point as the systems evolves. It was not necessary to create a new recursive approximation for the last estimation stage; nevertheless, as future work, it would be interesting to analyse this second recursion and test if there is some improvement.

Finally, we considered that the proposed method is adequate to estimate coefficients by a complex system, affecting, positively, the Equivalent Artificial Neural Network (EANN) or, in other words, a MISO system.
